# Exercise Ameliorates Insulin Resistance of Type 2 Diabetes through Motivating Short-Chain Fatty Acid-Mediated Skeletal Muscle Cell Autophagy

**DOI:** 10.3390/biology9080203

**Published:** 2020-08-03

**Authors:** Ling Yang, Haiqi Lin, Wentao Lin, Xiaoyang Xu

**Affiliations:** 1 National Demonstration Center for Experimental Sports Science Education, School of Physical Education, South China Normal University, Guangzhou 510006, China; yangling2018010077@126.com; 2 School of Physical Education, Shao Guan University, Shaoguan 512000, China; 3 School of Physical Education, South China University of Technology, Guangzhou 510641, China; sphqlin@scut.edu.cn; 4 Guangzhou Institute of Physical Education, Guangzhou Sport University, Guangzhou 510500, China; gtwtlin@163.com

**Keywords:** exercise, diabetes mellitus, skeletal muscle cells, insulin resistance, short-chain fatty acids, autophagy

## Abstract

**Background**: Exercise can ameliorate type II diabetes mellitus (T2DM) by regulating intestinal flora metabolites. However, the detailed mechanism needs to be further explored. **Methods**: A T2DM model using mice was established by feeding them a high-fat diet and giving them subsequent streptozocin injections. Fasting blood glucose and serum insulin were determined by blood glucose meter and radioimmunoassay, respectively. Intestinal flora was measured by 16sRNA sequencing. SCFA content was measured by gas chromatography (GC) or enzyme-linked immunosorbent assay (ELISA). A fluorescently labeled 2-deoxyglucose (2-NBDG) kit was employed to detect glucose uptake capacity, and western blot was utilized to explore the signaling pathway of insulin resistance and cell autophagy. **Results**: In the T2DM model, along with a reduction in insulin resistance (IR), exercise reversed the decline of intestinal Bacteroidetes and the increase of Firmicutes. For metabolites of *Bacteroides*, exercise restored the decline in total intestinal and plasma short-chain fatty acids (SCFAs) in T2DM mice. However, the administration of GLPG0974—the inhibitor of G protein-coupled receptor 43 (GPR43), which is the receptor of SCFAs—abolished exercise-mediated alleviation in IR in vivo and acetate-mediated reduction of skeletal muscle IR (SMIR) in vitro. Mechanistically, exercise induced skeletal muscle cell autophagy, thereby ameliorating SMIR, which was neutralized by GLPG0974 exposure. **Conclusions**: Exercise-mediated SCFAs-upregulation may ameliorate insulin resistance (IR) through increasing autophagy of skeletal muscle cells by binding to GPR43. This study provides a theoretical basis for targeting gut bacterial metabolites to prevent T2DM.

## 1. Introduction

Diabetes mellitus (DM) is a serious chronic metabolic disease caused by a relative or absolute deficiency in insulin secretion. The incidence of DM, especially type II diabetes mellitus (T2DM), has recently increased worldwide [1,2] and is expected to double by 2030, making it the seventh leading cause of death worldwide [3]. When liver, muscle and adipose tissues are less sensitive to insulin, it will cause relative insulin resistance (IR). Pancreatic β cells become dysfunctional due to the increased load and cannot compensate for IR, thus inducing T2DM [4]. Therefore, mitigating IR to reduce the burden on pancreatic β cells is the most effective way to delay T2DM progress [5]. To date, it is believed that IR runs through the natural course of T2DM, and the resulting hyperinsulinemia can easily promote the occurrence of diseases such as hypoadiponectinemia and cardiovascular disease, but a complete treatment strategy is yet to be realized [6,7]. It is of great significance to study the specific mechanism of IR in DM pathogenesis and the related pathophysiological changes in the human body to improve insulin sensitivity, protect islet β cells and reduce the risk of T2DM complications.

Although T2DM is associated with a genetic predisposition, it is unlikely that the gene pool will significantly change in a short period of time [8]. Therefore, the increase in the incidence of T2DM may be related to lifestyle factors rather than genetic factors. Accumulating evidence has elucidated that long-term, moderate physical activity including aerobic and voluntary exercises, representing habitual exercise, contribute to glucose uptake and fat oxidation [9]. Additionally, the long-term benefits of exercise therapy for T2DM have been recognized [10]. Exercise facilitates the decline of blood branched-chain amino acid [11], thereby promoting glucose and lipid metabolism in patients with T2DM [12]. Exercise also increases insulin sensitivity and reduces IR in T2DM by mediating the competing endogenous RNAs (CeRNAs) axis [13,14]. Swimming can suppress high-fat diet (HFD)-induced IR by decreasing liver and muscle lipid accumulation and modulating energy metabolism in skeletal muscles [15]. IR usually begins with reduced insulin sensitivity in skeletal muscle cells of T2DM [16]. Previous studies demonstrated that regular exercise can enhance glucose oxidation, improving glucose uptake and skeletal muscle insulin resistance (SMIR) by increasing transcription of glucose transporter 4 (GLUT4), a key glucose transporter subtype in skeletal muscle responsible for insulin- and exercise-stimulated glucose transport [17]. Accumulating evidence has indicated that Ca^2+^ signaling and the κB (IκB)/nuclear factor κB (NFκB) inflammatory pathway account for the mechanism by which exercise ameliorates glucose homeostasis and SMIR in individuals with T2DM [18,19]. The above studies illustrate the important role of exercise in treating T2DM by changing the SMIR. However, the specific molecular mechanism underlying exercise-mediated improvements in SMIR needs to be further explored in T2DM.

Exercise impacts the intestinal flora community and metabolites [20]. Early life exercise may promote lasting brain health and metabolic homeostasis through regulating gut bacterial metabolites [21]. Exercise has been proved to increase Firmicutes species and decrease *Bacteroides/Prevotella* spp. in both normal and diabetic mice [22]. In comparison with patients undergoing moderate aerobic exercise, there were more Bacteroidetes and decreased Firmicutes in obese adults who had received severe aerobic exercise for 10 weeks [23]. Exercise is considered a metabolism modulator. The metabolic changes, such as purine metabolites, tryptophan metabolites, as well as metabolites from the gut microbiota, blood, urine, and feces induced by running may be partially responsible for the running-related promotion of health [24,25]. Long-distance endurance running can immediately cause striking metabolic changes (a total of 40 fecal metabolites), such as organic acids and nucleic acid components, in the gut environment [26]. Short-chain fatty acids (SCFAs), including acetic acid, propionic acid, butyric acid and valeric acid, are the end metabolites of fermentation of dietary fiber by anaerobic intestinal microbiota [27], and their contents are also regulated by exercise. Autonomous running can increase the concentration of n-butyrate in the cecum of rats [28]. Increased butyrate production is also found in individuals with higher levels of aerobic fitness [29]. SCFAs have been confirmed to exert beneficial effects on atherosclerotic cardiovascular diseases [30] and obesity [31]. Research has shown that SCFAs biosynthesis and branched-chain amino acid catabolism in prediabetic patients who respond to exercise are enhanced [32], implying the involvement of intestinal SCFAs during exercise mediated the improvement on T2DM. However, the specific mechanism by which exercise affects T2DM through modulating SCFAs remains unclear.

Therefore, in this study we aimed to investigate whether exercise intervention-mediated reductions of T2DM symptoms involved SCFAs. It was found that exercise mediated the elevation of intestinal and plasma acetic acid, which thereby promoted autophagic events in skeletal muscle cells via binding to the G protein-coupled receptor 43 (GPR43), resulting in the enhancement of insulin sensitivity. These findings contribute to the development of new therapeutic options targeting SCFAs for T2DM.

## 2. Materials and Methods

### 2.1. Animal Model and Treatment

Animal experiments were carried out with approval by the Ethics Committee of South China Normal University and conducted in accordance with the China Code of Practice for the Care and Use of Animals for Scientific Purposes (SCNU-KY2017-077). The authors are accountable for all aspects of the work in ensuring that questions related to the accuracy or integrity of any part of the work are appropriately investigated and resolved. All animals had free access to food and water. During the experiment, food intake was recorded daily, and body weight was recorded weekly. After fasting for 12 h every week, blood samples were taken from the tail vein to detect fasting blood glucose. The animals were housed in a temperature-controlled (25 ± 2 °C) room with a 12 h light/dark cycle and relative humidity of 50%–70%. A total of 60 clean 4-week-old male C57Bl/6 J wild-type (WT) mice (SLAC, Shanghai, China) with a weight of 20–22 g were used to establish the T2DM model.

T2DM mice were modeled and properly adjusted according to the methods of previous literature [33]. All mice were randomly divided into a control group (*n* = 10), T2DM group (DM, *n* = 40). The control group was fed with common feed, and the DM group and the DM+Ex group were fed with a high-fat feed (HFD) (10% lard, 20% sucrose, 2.5% cholesterol, 1% sodium cholate, 66.5% ordinary feed) for four consecutive weeks. After 4 weeks of HFD feeding and 12 h of fasting, the DM group and DM+Ex group were injected intraperitoneally with 25 mg/kg of streptozocin (STZ) (S0130, Sigma, Saint Louis, MO, USA) for 5 consecutive days. The control group was intraperitoneally injected with isometric citric acid buffer solution. The blood glucose of the mice in each group was measured 72 h after the final injection. When blood glucose exceeded 16.7 mmol/L, it was considered a successful model of T2DM. Twenty mice with T2DM were selected and randomly divided into two group including DM (*n* = 10) and T2DM combined with exercise intervention group (DM+Ex, *n* = 10). Then, the control group and the DM group were fed with normal diets, and the mice in the DM+Ex group received an aerobic exercise intervention using an improved Ploug training program. In brief, mice were first subjected to 2 adaptive swimming training sessions of 10 min each. The first 3 days of training lasted 20 min, 30 min and 45 min, respectively. From the fourth day, the swimming time increased to 60 min every day. The training program was 5 days per week and the training cycle was 8 weeks. Furthermore, GLPG0974 (HY-12940, MCE, Monmouth, NJ, USA) was orally dosed as a single esophageal gavage at 10 mg/kg to DM mice before the exercise intervention at the fifth week for 8 consecutive weeks (once per week).

### 2.2. Detection of Fasting Blood Glucose and Fasting Insulin

During the process of establishing the model, blood glucose was measured once per week. After fasting for 12 h, blood was collected from the tail vein and the concentration of blood glucose in individual mice was measured using a OneTouch Ultra blood glucose meter (LifeScan, Milpitas, Santa Clara, CA, USA). For the measurement of serum insulin, blood was collected from the internal iliac vein at the end of the experiment, and the plasma was centrifuged (3500 rpm for 10 min) at 4 °C and the supernatant was collected. Then, the insulin level in serum was measured by radioimmunoassay.

### 2.3. Oral Glucose Tolerance Test (OGTT) and Insulin Tolerance Test (ITT)

In brief, mice in each group were fasted for 16 h before taking the OGTT measurement. D-(+)-Glucose (G8270, Sigma, Saint Louis, MO, USA) was dissolved in diH_2_O (F0020, Solarbio, Peking, China) and orally administered to the fasted mice (2 g/kg of body weight) using a 20-gauge stainless steel gavage feeding needle (Fisher Scientific, Waltham, MA, USA). Whole blood samples (2–3 μL each) were collected from a tail-clip bleed immediately before glucose administration and 15, 30, 60, 90 and 120 min after glucose administration. Then, blood glucose was measured using a OneTouch Ultra blood glucose meter and the area under the glucose tolerance curve was determined by Graph Pad Prism 7.0 software. For ITT, after fasting for 6 h, mice were injected intraperitoneally with 0.75 units/kg of human regular insulin (Eli Lilly, Indianapolis, IN, USA). Subsequently, the concentrations of blood glucose were measured before insulin injection and at 15, 30, 60, 90 and 120 min post-injection. The area under the insulin tolerance curve was calculated by Graph Pad Prism 7.0 software.

### 2.4. Gut Microbiome Analysis by 16s RNA Sequencing

At the end of the experiment, mice were fed separately after training, and 1.5 g feces from each mouse was collected. The frozen fecal samples were batched for microbiome assessment using 16S ribosomal RNA (rRNA) gene V3-V4 region-based sequencing. Briefly, genomic DNA was extracted from the mixed feces by the QIAamp DNA stool mini kit (Qiagen, Dusseldorf, Germany) according to the manufacturer’s instructions. The extracted DNA was sub-packaged into multiple tubes to avoid multi-gelation and stored at −20 °C. The V3-V4 region of the bacterial 16S rRNA gene was amplified using PCR with the TaKaRa Ex Taq HS Kit (TaKaRa Bio, Shiga, Japan) by Novogene biotechnology technology (Tianjin, China).

### 2.5. Determination of SCFAs

At the end of the experiment, mice were fed separately after training, and 1.5 g feces of each mouse was collected for the detection of SCFA contents. SCFAs in feces were determined using gas chromatography (GC). First, 50 mg of lyophilized stool samples were put into a round-bottomed flask and gently suspended in 2 mL of distilled water. Subsequently, 0.4 mL of 50% aqueous sulfuric acid and 2 mL of diethyl ether were added and mixed with an orbital shaker for 1 h, and then centrifuged at 3000 rpm for 10 min. Anhydrous CaCl_2_ was added to the collected supernatant to remove residual water, then 2 μL supernatant was analyzed by injection in the GC system (Agilent 7890A gas chromatograph fitted with a flame ionization detector). SCFAs were quantified by an external standard method using the mix standard solution of acetic, propionic, isobutyric, butyric, isovaleric, valeric acids. For the measurement of plasma SCFAs, a mouse SCFAs ELISA Kit (BOS22873, BOSK Bio, Wuhan, China) was used to quantify the total SCFAs in mouse plasma. After forming an antibody–antigen–enzyme labeled antibody complex, the substrate ethyl benzylbenzidine (TMB) was added to develop color. Then, the absorbance (OD value) was measured with a microplate reader (SpectraMax 190, MD, San Francisco, CA, USA) at a wavelength of 450 nm.

### 2.6. Isolation and Culture of Primary Skeletal Muscle Cells

The muscles of the sacrificed mice were collected in a petri dish. After washing 3 times with Hank’s buffer (14185052, Thermo Fisher, Waltham, MA, USA), the fat and connective tissue were removed, and the muscle specimens were cut into 1 mm^3^ cubes. After washing 3 times by Hank’s, the upper liquid and floating tissues were discarded after standing for 1 min. Trypsin (SH30042, Hyclone, Logan, UT, USA) was added to digest muscle tissue at 37 °C for 20 min. After stopping the digestion using 10% fetal bovine serum (FBS) (10091-148, Gibco, Carlsbad, CA, USA) samples were sieved using 100, 200 and 400-mesh sieves and the filtrate was collected and centrifuged at 1000 rpm for 10 min. After discarding the supernatant, the cells were resuspended in MEM (SH30265, Hyclone, Logan, UT, USA) containing 10% FBS (10091-148, Gibco, Carlsbad, CA, USA) and 1% penicillin/streptomycin (15070-063, Gibco, Carlsbad, CA, USA). The suspension was added to an uncoated flask. The fibroblasts were removed by differential adherence. After 1 h incubation at 37 °C, the cells were inoculated in L-polylysine-bottomed culture flasks (Corning, Corning city, NY, USA) and cultured for 4 days with a change of fluid once per day. Then, cells were used to perform the next experiments.

### 2.7. 2-NBDG Glucose Uptake Assays

Skeletal muscle cells were first grown in glucose free medium to mid log phase. The cells were harvested, resuspended in the aforementioned medium and incubated with 20-μM 2-NBDG (186689, APExBIO, Houston, TX, USA) at 30 °C for 30 min. The uptake reaction was stopped by washing the cells three times with 1×phosphate-buffered saline (PBS, pH 7.4). Live cells were visualized with an Olympus FluoView confocal microscope (FV1000, Tokyo, Japan) under a 63× oil-immersion objective lens using GFP filter. The quantification of 2-NBDG fluorescence was calculated using ImageJ v1.4r software (NIH). Briefly, the mean fluorescence intensity of individual cells within the field of view was calculated and normalized by subtracting the background fluorescence signal from a region without any cells. At least 200 cells were analyzed from three independent experiments.

### 2.8. Western Blot Analysis

The cellular proteins were extracted form skeletal muscle cells and lysed by RIPA lysate (R0010, Solarbio, Peking, China). Equal amounts of protein were electrophoresed on sodium lauryl sulfate polyacrylamide gel (SDS-PAGE) with a loading of 20 μL per well. The protein was then transferred to an activated PVDF membrane (FFP26, Beyotime, Shanghai, China) and blocked with 5% skim milk powder (P0216, Beyotime, Shanghai, China) for 2 h. Appropriately diluted primary antibodies against Beclin-1 (Abcam, Cat# ab223372), p62 (Abcam, Cat# ab101266), p-mTOR (Abcam, Cat# ab109268), mTOR (Abcam, Cat# ab2732), p-AKT^Ser473^ (Abcam, Cat# ab18206), p-IRS1^Ser616^ (CST, Cat# 2386S), IRS (CST, Cat# 2382S), AKT (CST, Cat# 2938S), LC3 (Affinity, Cat# AF5402) and β-actin (Abcam, Cat# ab8227) were added and incubated at 4 °C overnight. After washing the membrane, the corresponding secondary antibody (Cat# A0208 and A0216, Beyotime, Shanghai, China) was added for incubation. Chemiluminescence detection was then carried out using an enhanced chemiluminescence reagent (P0018AS, Beyotime, Shanghai, China). After development and fixing treatment, the film was photographed by a gel imaging analysis system (UniCel Dxl800, Beckman Coulter, Pasadena, CA, USA).

### 2.9. Statistical Analysis

Data were presented as mean ± standard deviation (SD) of at least three independent experiments and analyzed by GraphPad Prism 7.0 software (GraphPad Software, Inc., San Diego, CA, USA). Significant differences of treatments versus their respective controls were determined using the Student’s t-test for paired experiments or two-way ANOVA, where * *p* < 0.05 and ** *p* < 0.01 indicates a significant difference and extreme significant difference, respectively.

## 3. Results

### 3.1. Exercise Intervention Affects Intestinal Flora Distribution in T2DM

To evaluate the dynamics of the bacterial community in T2DM after exercise intervention, we first checked whether the model was established successfully. The results showed that the body weight in the DM group began to decrease slowly after STZ injection compared with the constant weight gain in the control group, whereas there was a slow increase in body weight after 8 weeks of exercise intervention for the DM-Ex group compared to the DM group (Figure S1A). Compared with the consistently lower fasting blood glucose level in the control group, the blood glucose maintained a high level (approximately 30 nM) after STZ injection in the DM group, while the STZ injection-mediated elevation of blood glucose was inhibited by the exercise intervention from weeks 9 to 13 (Figure S1B). The exercise intervention suppressed the elevated insulin levels in the DM group at the end of modeling (Figure S1C). Moreover, the increased glucose tolerance and insulin tolerance after the STZ injection were significantly restored in the DM+Ex group (Figure S1D–G). There were no differences in dietary intake between the groups for the first four weeks before STZ injection (Figure S1H). The above results indicate that the T2DM model was successfully established and the exercise intervention had a beneficial effect on some biochemical indicators of diabetes such as fasting blood glucose, insulin level, glucose tolerance and insulin tolerance.

**Figure 1:**
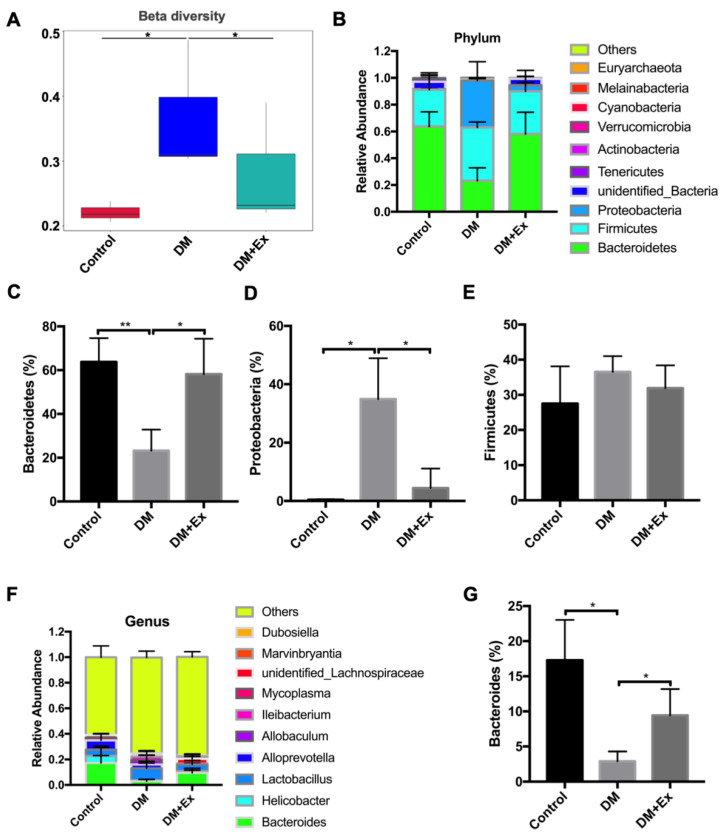
Exercise intervention affects intestinal flora distribution in mice with diabetes mellitus (DM). (**A**) Mice were randomly divided into three groups (control, DM and DM+Ex) and modeled. Beta diversity analysis was determined by 16srRNA sequencing; (**B**) analysis of all intestinal flora in different groups at the phylum level; (**C**–**E**) proportion of Bacteroidetes, Firmicutes and Proteobacteria in each group; (**F**) Analysis of intestinal flora in different groups at the genus level; (**G**) proportion of *Bacteroides* in each group. Control—normal mice; DM—diabetes mellitus; DM+Ex—diabetic mice treated with exercise. * *p* < 0.05. ** *p* < 0.01.

Then, 16srRNA sequencing was employed to detect the characteristics of the intestinal flora in different groups. The results showed that alpha diversity in the DM and DM-Ex groups was higher than the control group, but without any statistically significant changes (Figure S2A). The beta diversity in the DM group was significantly higher than the control group, while it was decreased markedly after exercise (Figure 3A). The genus-level evolutionary tree results indicated that the differences in bacteria between groups were mainly concentrated in Bacteroidetes and Firmicutes (Figure S2B). Indeed, examining the difference in distribution of intestinal flora at the phylum level, we found that the relative abundance of Bacteroidetes decreased significantly in the DM group, although the abundance significantly recovered after exercise (Figure 1B,C. Changes in Proteobacteria abundance were the reverse of those for *Bacteroidetes* (Figure 1D). No significant changes in Firmicutes abundance were observed among the three groups (Figure 1E). At the genus level, the results also revealed that *Bacteroides* abundance significantly decreased in the DM group, but increased after exercise (Figure 1F,G. These results suggest that an exercise intervention has a positive regulation effect on the disturbance of microflora in T2DM.

### 3.2. Exercise Reverses the Reduction of SCFAs in T2DM

**Figure 2:**
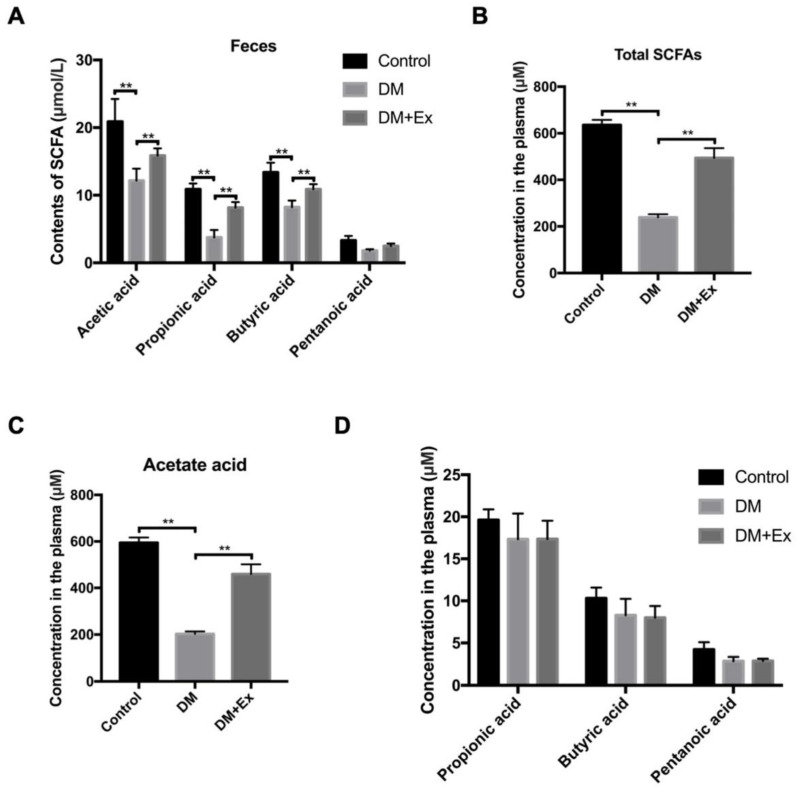
Exercise recovers a DM-mediated reduction of short chain fatty acids (SCFAs). (**A**) Detection of SCFAs in stool of control, DM and DM+Ex groups by gas chromatography (GC); (**B**) detection of total SCFAs concentration in plasma by ELISA; (**C**,**D**) concentration of acetate acid, propionic acid, butyric acid and pentanoic acid in the plasma of each group detected by GC. ** *p* < 0.01.

SCFAs are the main metabolites of *Bacteroides* and other members of the Bacteroidetes [34]. Since the differences in flora after the exercise intervention appeared in the abundance of *Bacteroides*, we speculated that the contents of SCFAs may be involved in this process. On the basis of GC analysis, the results demonstrated that the fecal acetic acid, propionic acid and butyric acid, which were the main components of SCFAs in the DM group, were significantly lower than those in the control group and pentanoic acid exhibited a statistically insignificant downward trend, while the reduction of the above SCFAs was obviously recovered by exercise management (Figure 2A). Meanwhile, after digestion and absorption of intestinal SCFAs in colonic epithelial cells, some enter the blood circulation [35]. SCFAs that enter different systemic circulations can affect cardiovascular metabolism, regulate fat, skeletal muscle and liver function and improve blood glucose and insulin sensitivity [36]. Therefore, we determined the changes in plasma SCFAs. Total plasma SCFAs content was significantly reduced by 67% in the DM group, whereas it was notably restored after exercise (Figure 2B). According to the analysis of mass spectrometry results, we observed that the decreased plasma SCFAs in DM group was mainly caused by a sharp decline in acetic acid, but not propionic acid, butyric acid and valeric acid (no significant difference) (Figure 2C,D. The above results demonstrate that exercise improves the DM-mediated decline in SCFAs, mainly by adjusting the content of plasma acetic acid.

### 3.3. The Block of GPR43 Abolishes Exercise-Mediated Improvements of Insulin Resistance in T2DM

**Figure 3:**
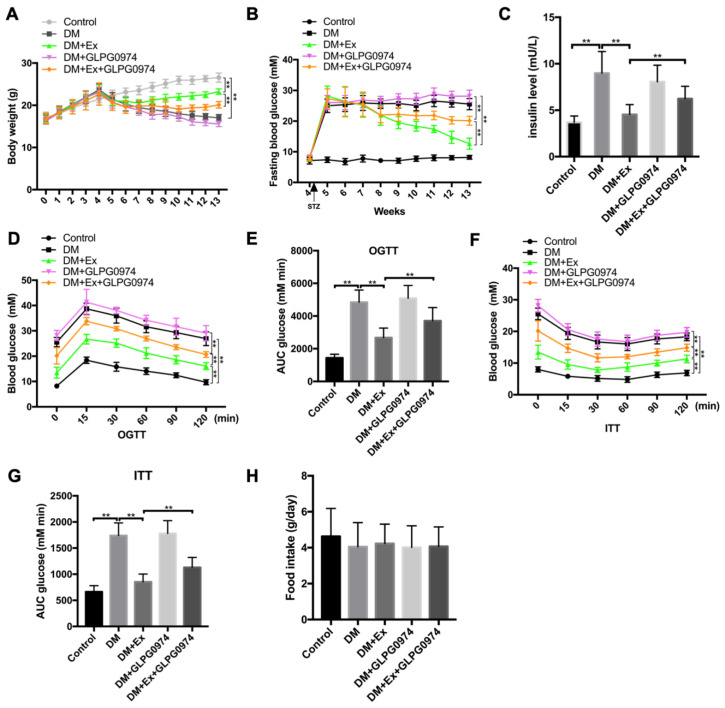
GPR43 antagonist inhibits the improvement of DM symptoms by exercise. (**A**) After the T2DM model was established, all diabetic mice were challenged with GLPG0974 and the body weight of mice in each group was measured; (**B**) fasting blood glucose in each group were determined by a OneTouch Ultra blood glucose meter; (**C**) insulin levels in mice of each group as detected by ELISA kit; (**D**) fasting blood glucose was performed after glucose administration for 15, 30, 60, 90 and 120 min and the curve of oral glucose tolerance test (OGTT) was made; (**E**) area under glucose tolerance curve in each group; (**F**) fasting blood glucose level was determined after insulin injection for 15, 30, 60, 90 and 120 min and a tolerance test (ITT) was created; (**G**) area under the insulin tolerance curve in different groups; (**H**) food intake in control mice, diabetic mice treated with or without exercise and diabetic mice treated with or without GLPG0974. ** *p* < 0.01.

The SCFA-mediated activation of free fatty acid receptor-2/G and protein-coupled receptor 43 (FFA2/GPR43) affects insulin signaling and GPR43 deficiency can lead to decreased insulin sensitivity [37]. We found that exercise promoted fecal and plasma SCFAs and ameliorated IR. We speculated that exercise-induced IR may involve the regulatory axis of SCFAs/GPR43. Therefore, we blocked the interaction of SCFAs and GPR43 with antagonists (GLPG0974) in the T2DM model to test this hypothesis. Although the GPR43 transcriptional level in the intestinal tract was not affected by STZ injection and/or exercise intervention (Figure S3A), the results of the RT–PCR assay showed that exercise exposure significantly elevated the expression of GPR43 in skeletal muscle of diabetic mice, and the administration of GPR43 antagonist GLPG0974 not only inhibited the GPR43 level in diabetic mice, but abolished the exercise-mediated promotion effect of GPR43 expression (Figure S3B). As shown in Figure 3, in comparison to the control group, body weight began to drop from 5 weeks and continued to decline for eight weeks consecutively in the DM group (gray line vs. black line), and the decline of body weight was reduced by exercise treatment (green line vs. black line). However, the addition of GLPG0974, an antagonist of GPR43, inhibited the exercise-mediated weight gain in diabetic mice (green line vs. yellow line) (Figure 3A). In addition, the exercise-initiated improvement in fasting blood glucose in diabetic mice was also reversed by the administration of GLPG0974 from weeks 9 to 13 (green line vs. yellow line) (Figure 3B). At the end of modeling, serum insulin levels were elevated in the DM group. Although exercise exposure inhibited serum insulin content, blocking GPR43 using GLPG0974 significantly abrogated the effects of exercise in diabetic mice (Figure 3C). On the basis of OGTT and ITT assays, the exercise-mediated decrease in glucose tolerance (Figure 3D,E and insulin tolerance (Figure 3F,G in diabetic mice was also reversed by the administration of GLPG0974, and the treatment of the antagonist alone had little effect on those two indicators. Additionally, food intake was consistent between groups (Figure 3H). These results indicate that blocking SCFAs/GPR43 signal transduction attenuates the effect of exercise on IR and suggests that exercise-mediated changes may involve this signal.

### 3.4. GPR43 Antagonist Interrupts Exercise and SCFAs-Mediated Amelioration in SMIR

**Figure 4:**
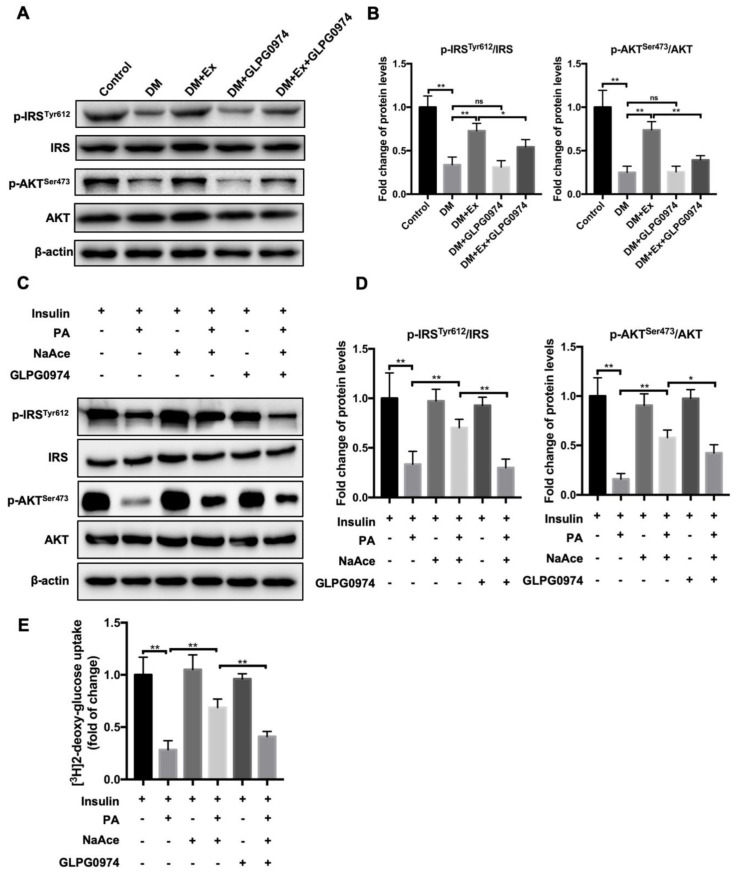
GPR43 antagonist blocks exercise-mediated amelioration in skeletal muscle IR (SMIR). (**A**) Primary skeletal muscle cells were isolated from the mice of different groups including control, DM, DM+Ex, DM+GLPG0974 and DM+Ex+GLPG0974. Then p-IRS^Tyr612^, IRS, p-AKT^Ser473^ and AKT, key proteins of insulin signaling pathway were determined by western blot; (**B**) semiquantitative statistics of p-IRS^Tyr612^ and p-AKT^Ser473^ normalized to total IRS and AKT, respectively; (**C**) primary skeletal muscle cells were isolated from wild-type mice. The insulin resistance skeletal muscle cells were established by the addition of palmitate (PA). Expression of p-IRS^Tyr612^, IRS, p-AKT^Ser473^ and AKT were detected by western blot; (**D**) semiquantitative statistics of p-IRS^Tyr612^ and p-AKT^Ser473^ normalized to total IRS and AKT, respectively; (**E**) glucose uptake capacity of in each group measured by fluorescently labeled 2-deoxyglucose (2-NBDG). * *p* < 0.05. ** *p* < 0.01.

SMIR is a central feature of T2DM and is defined as a subnormal response of tissues to insulin action and exercise treatment, resulting in decreased glucose uptake ability in skeletal muscle [38]. Next, we measured the detection of SMIR after exercise exposure. In primary skeletal muscle cells isolated from the mice of different groups, we discovered that IR increased in the DM group compared to the control group, which was manifested in a significant reduction of phosphorylation activity of IRS at the site of Tyr612 (p-IRS^Tyr612^) and phosphorylation activity of AKT at the site of Ser473 (p-AKT^Ser473^) (Figure 4A,B. In the skeletal muscle cells from DM-Ex group, the expression of p-IRS^Tyr612^ and p-AKT^Ser473^ was significantly reactivated compared to the DM group. Once the GLPG0974 was administrated in diabetic mice, the activities of p-IRS^Tyr612^ and p-AKT^Ser473^ were not affected, while the exercise-initiated reactivation of p-IRS^Tyr612^ and p-AKT^Ser473^ were notably limited by the treatment of GLPG0974 in diabetic mice (Figure 4A,B. Next, we isolated primary skeletal muscle cells from WT mice to explore the effects of GPR43 antagonists on IR in vitro and to investigate whether SCFA (acetic acid) was involved in the process. The insulin resistance skeletal muscle cells were established by the addition of palmitate (PA). As shown in Figure 4B, PA treatment significantly induced the decrease in the expression of p-IRS^Tyr612^ and p-AKT^Ser473^ in skeletal muscle cells, which suggested that the IR model was successfully established (Figure 4C,D. The reduction in the activities of p-IRS^Tyr612^ and p-AKT^Ser473^ was significantly alleviated after sodium acetate treatment. However, it was found that the remission effect of sodium acetate on IR was suppressed by the exposure of GPR43 antagonists (along with the decrease in the activities of p-IRS^Tyr612^ and p-AKT^Ser473^) (Figure 4C,D. Additionally, we also determined the glucose uptake capacity in different skeletal muscle cells using fluorescent-labeled 2-deoxyglucose (2-NBDG) kit. The results showed that sodium acetate significantly restored the PA-induced reduction in capacity for sugar uptake, whereas the addition of GPR43 antagonists inhibited the promotion role of sodium acetate on sugar uptake (Figure 4E). Next, we investigated whether the addition of GLPG0974 would affect the contents of fecal and blood SCFAs in diabetic mice. The results showed that both intestinal and plasma SCFAs were affected by the administration of GLPG0974 in diabetic mice who received the exercise treatment, that is, the blocking of GPR43 did not affect the exercise-mediated elevation of SCFAs including acetic acid (Figure S4A,B). These results confirmed that exercise and acetate-mediated amelioration of SMIR are dependent on GPR43 since exercise can increase blood acetic acid content and improve SMIR. It is possible that the SCFAs/GPR43 signal axis is involved in exercise-mediated improvements in SMIR.

### 3.5. SCFAs-Mediated the Remission in SMIR Is in a Autophagy Dependent Manner

**Figure 5:**
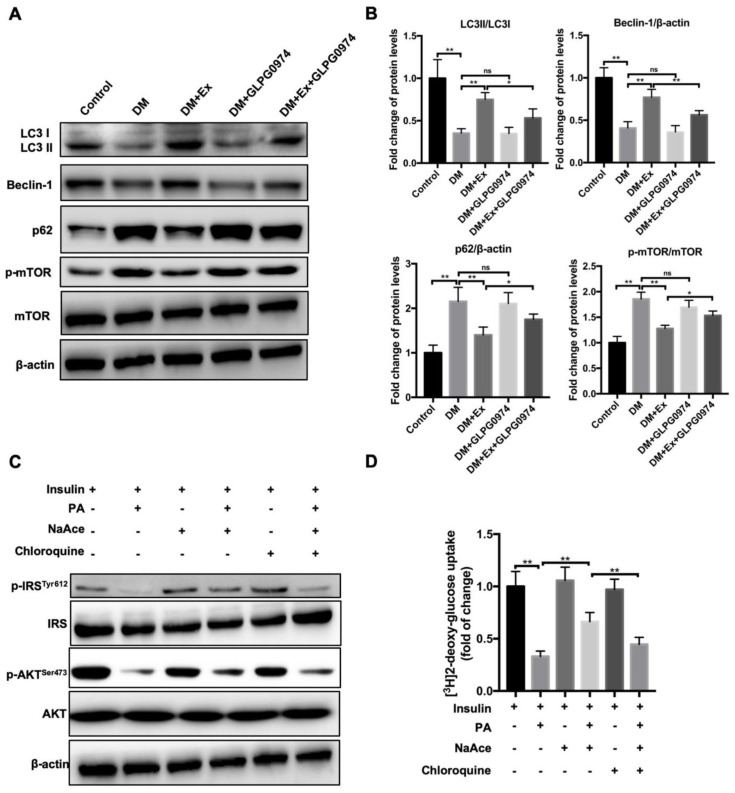
Exercise-mediated SCFAs changes ameliorate IR mainly by increasing autophagy in skeletal muscle cells. (**A**) Primary skeletal muscle cells were isolated from the mice of different groups including control, DM, DM+Ex, DM+GLPG0974 and DM+Ex+GLPG0974. The expression of autophagy-related proteins p62, LC3, Beclin1, p-mTOR and mTOR in different groups was analyzed by western blot; (**B**) quantitative analysis of the ratio of LC3II/LC3I, p-mTOR, Beclin 1 and p62 in the left panel (**A**); (**C**) primary skeletal muscle cells were isolated from wild type mice. The insulin resistance skeletal muscle cells were established by the addition of palmitate (PA). Autophagy flux was blocked by the administration of chloroquine. The p-IRS^Tyr612^, IRS, p-AKT^Ser473^ and AKT expression in different treatment groups were determined by western blot; (**D**) glucose uptake capacity of cells in each group measured by fluorescently labeled 2-deoxyglucose (2-NBDG). * *p* < 0.05. ** *p* < 0.01.

The enhancement of skeletal muscle cell autophagy was considered to be a critical switch to ameliorate SMIR [39]. Therefore, we next assessed whether autophagic events participated in the process of exercise-mediated the improvement of SMIR. The detection data of autophagy-related proteins revealed that exercise significantly increased LC3II/LC3I and Beclin 1 in the DM group compared with the control group and markedly reduced the enhanced p62 and p-mTOR/mTOR (Figure 5A,B. However, the addition of the antagonist inhibited the expression of these proteins, specifically reflected in the decreased expression of LC3II/LC3I and Beclin 1 and the elevated expression of p62 and p-mTOR/mTOR (Figure 5A,B. The data indicated that exercise promoted autophagy flux in skeletal muscle cells, which may have contributed to the elevation of insulin sensitivity. However, whether SCFAs were involved in this process remains unclear. Thus, we determined the role of autophagy on SCFAs-mediated the improvement of SMIR using normal primary skeletal muscle cells. In agreement with the above data, sodium acetate treatment significantly alleviated the increase of PA-induced IR by activating the activities of p-IRS^Tyr612^ and p-AKT^Ser473^ (Figure 5C). In contrast, the addition of autophagy inhibitor chloroquine notably inhibited the alleviation of IR caused by sodium acetate exposure (Figure 5C and Figure S5A,B). Additionally, the glucose uptake ability of the cells treated with sodium acetate upregulated the PA-induced reduction in sugar uptake, but the addition of chloroquine halted the promotion of sodium acetate and the capacity of glucose uptake by skeletal muscle cells (Figure 5C). These results demonstrate that acetate improves the IR of skeletal muscle cells depending on cell autophagy. Perhaps exercise can increase plasma acetic acid levels and acetic acid, through its receptor GPR43, activates autophagy in skeletal muscle cells, thereby improving the IR of skeletal muscle cell.

## 4. Discussion

Increasing evidence indicates that exercise can improve IR by regulating the intestinal flora and its metabolites in T2DM. An exercise intervention improves glucose homeostasis and insulin sensitivity in patients with pre-diabetes, which is closely related to changes in intestinal flora and its ability to ferment proteins and carbohydrates [32]. Whole body vibration changes the composition of intestinal microflora, slightly reduces alpha diversity, increased the number of SCFAs-producing bacteria and elevates the number of *Bacteroides* and *Alispes* in T2DM mouse [40]. Evidence shows that exercise regulates the concentration of SCFAs in lean and overweight healthy subjects and diabetic patients [31]. This suggests that increased SCFAs production through microbiota profile changes may be a potential mechanism for physical exercise to improve DM. The present study demonstrated that exercise intervention ameliorated insulin sensitivity by increasing fecal and blood SCFAs, primarily via the elevation of fecal and blood acetic acid, thereby binding to GPR43 and activating autophagy of skeletal muscle cells. The findings imply that SCFAs are the critical regulators during the exercise-mediated remission of IR in T2DM.

Targeting intestinal flora is an important channel in determining exercise to improve glucose metabolism and insulin sensitivity. The intestinal flora in obese diabetic mice is significantly different from that of normal mice, as the former has a higher percentage of *Lactobacillus spp.* and lower percentages of order Bacteroidales and family Lachnospiraceae [41]. The β-diversity and relative abundance of intestinal microflora inT2DM mice changes significantly, for example, *Bacteroides* and *Pretium* are reduced at the family and genus levels [42]. Evidence shows that the intestinal flora and its metabolites can regulate the hormone secretion function of intestinal endocrine cells, thereby regulating appetite and insulin secretion, which is considered to be the basis of the intestinal flora’s effect on many metabolic diseases (e.g., obesity and type II diabetes) [43]. The exercise-mediated improvement of T2DM may be attributed to the ability of the intestinal flora in exercise responders to improve the function of the intestinal barrier [44]. Exercise, as a therapeutic intervention for the prevention and treatment of IR, has been confirmed [45]. This study confirmed that exercise not only relieved IR, but also decreased the diversity of intestinal bacteria and increased the percentage of *Bacteroides* in diabetic mice compared to control mice. Gut commensal *Bacteroides* can prevent obesity and ameliorate IR in lean phenotypes in Atg7^ΔCD11c^ mice by improving metabolic disorders [46]. It is possible that exercise regulates IR mainly by changing the abundance of *Bacteroides*. However, further analysis of intestinal bacteria composition differences at the species level is required.

SCFAs are the final products of fermentation by intestinal bacterial (mainly *Bacteroides*) and are also thought to be involved in the development of intestinal motility and intestinal diseases [47]. The beneficial effects of exercise are related in part to the alteration of the intestinal microbiota and subsequent changes in the intestinal SCFA profile, especially changes in acetate [48] and butyrate concentration [28,29]. Additionally, exercise can reduce the intestinal transit time, which is inversely correlated with the fecal n-butyrate concentration [49]. Several animal studies have consistently demonstrated the metabolic benefits of butyrate or propionate treatment in energy expenditure and glucose homeostasis [50,51]. SCFAs have also been proved to play a beneficial role in improving DM [52]. For example, in overweight men with prediabetes, species falling into the *Bacteroides* genus and order Clostridiales, most of which are involved in the production of SCFAs, underwent a significant strain-level genomic variation by exercise [32]. A high fiber diet selectively promotes a group of SCFAs producers as the major active producers to alleviate DM [53]. Consistent with a previous report, our study also revealed that exercise upregulated the reduction of total plasma SCFAs in DM mice. Previous evidence has shown that acetic acid improves muscle glucose uptake, reduces hyperglycemia in diabetic mice and its derivatives are confirmed to treat IR and T2DM [54,55]. Of note, the changes we presented in total plasma SCFAs were mainly highlighted in plasma acetic acid. GPR43, as a SCFA receptor, has a unique response to intestinal flora-derived nutrients (e.g., acetate, propionate and butyrate) [56]. SCFAs improve the metabolic function of T2DM through FFAR2/GPR43 and FFAR3 (or GPR41) (including control of blood glucose levels, IR and GLP-1 secretion) [57]. We found that GPR43 inhibition significantly blocked the improvement of DM symptoms by exercise. The above results illustrate that improved IR mediated by exercise may be related to SCFAs, especially acetic acid and downstream GPR43 signaling axes. However, there are still some doubts about this: First, why does exercise only alter plasma acetic acid levels? Second, what is the mechanism by which exercise regulates intestinal SCFAs (acetic acid) into the bloodstream? Moreover, is this related to intestinal permeability? Is it possible that exercise regulates the intestinal epithelial cells to make the absorption capacity of acetic acid stronger, leading to the significant change in acetic acid, but not others? Are the differences related to exercise type? These questions still need to be addressed.

Acute exercise prevents fatty acid-induced SMIR by increasing triglyceride synthesis in skeletal muscle [58]. The skeletal muscle inflammatory pathway associated with IR in subjects with T2DM can be reversed by exercise [19]. Aerobic exercise may prevent IR by correcting a mismatch between fatty acid uptake and fatty acid oxidation in skeletal muscle [59]. Endurance exercise training increases insulin responsiveness through IRS/phosphatidylinositol 3-kinase (PI3 K)/Akt pathway [60]. Activities of Akt1 and glycogen synthase and phosphorylation of AS160 are increased with exercise, whereas activity of PI3 K associated with IRS-1 is reduced [61]. Fatty acids, such as gymnemic acid and palmitate, stimulate glucose uptake and alleviate inflammation and IR via PPARδ- and NFκB-mediated pathways and PI3K/AMPK/Akt pathways [62]. However, whether the improvement of SMIR by exercise is related to SCFA/GPR43 pathways is unclear. Our results indicated that GPR43 antagonists blocked exercise or sodium acetate-mediated improvements of IR in skeletal muscle tissue of diabetic mice by blunting insulin signaling transduction via inhibition of the activities of p-IRS^Tyr612^ and p-AKT^Ser473^. Since exercise also induced the elevation of plasma acetic acid in diabetic mice, we thought that the enhancement of insulin sensitivity induced by exercise could be associated with the acetic acid-mediated activation of insulin signaling. Previous studies have suggested that autophagy inhibition can promote SMIR, many of which are involved in the AMPK signaling pathway [39,63]. Exercise can increase AMPK activity in skeletal muscle, which in turn improves glucose uptake and insulin sensitivity [64]. Here, we observed that exercise induced the elevation of autophagy flux in skeletal muscle cells, while this change was GPR43 dependent. Additionally, sodium acetate-induced the remission on insulin resistance was abolished when autophagy was blocked. Possibly, exercise-mediated SCFAs changes (mainly acetic acid) improved SMIR by increasing autophagy in skeletal muscle cells. Whether AMPK signaling was involved in the exercise-induced elevation of plasma acetic acid and subsequent increase of autophagy in skeletal muscle cells remains unclear. Furthermore, the underlying mechanism in the process of the SCFAs-mediated improvement of SMIR needs clarification via microbiota transplantation such as *Bacteroidetes,* SCFAs or sodium acetate.

## 5. Conclusions

In summary, exercise management alleviates IR and is accompanied by significant changes in the distribution of the intestinal flora, specifically manifested in the reversal of attenuated Bacteroidetes and enhanced Proteobacteria in diabetic mice. Exercise mediated the increase of SCFAs, mainly of plasma acetic acid, improving IR by combining with GPR43, thereby promoting autophagy of skeletal muscle cells. Our findings provide new ideas for targeting SCFAs to treat T2DM.

## Data Availability

All data generated or analyzed during this study are included in this published article.

## Supplementary Materials

The following are available online at https://www.mdpi.com/2079-7737/9/8/203/s1.

## Abbreviations

T2DM	type II diabetes mellitus
SCFAs	short-chain fatty acids
GC	gas chromatography
2-NBDG	fluorescently labeled 2-deoxyglucose
GPR43	G-protein-coupled receptor 43
SMIR	skeletal muscle insulin resistance
IR	insulin resistance
CeRNAs	competing endogenous RNAs
HFD	high-fat diet
GLUT4	glucose transporter 4
NFκB	nuclear factor κB
STZ	streptozocin
OGTT	oral glucose tolerance test
ITT	insulin tolerance test
rRNA	ribosomal RNA
OUT	operational taxonomic unit
TMB	ethyl benzylbenzidine
