# Supplementary material for: Exercise Ameliorates Insulin Resistance of Type 2 Diabetes through Motivating Short-Chain Fatty Acid-Mediated Skeletal Muscle Cell Autophagy
Source: Biology (Basel). 2020 Aug 3;9(8):203. doi: 10.3390/biology9080203 (PMC7464264; doi:10.3390/biology9080203)
Supplement: Supplementary file 1 [file biology-09-00203-s001.zip › biology-874432-supplementary.pdf]

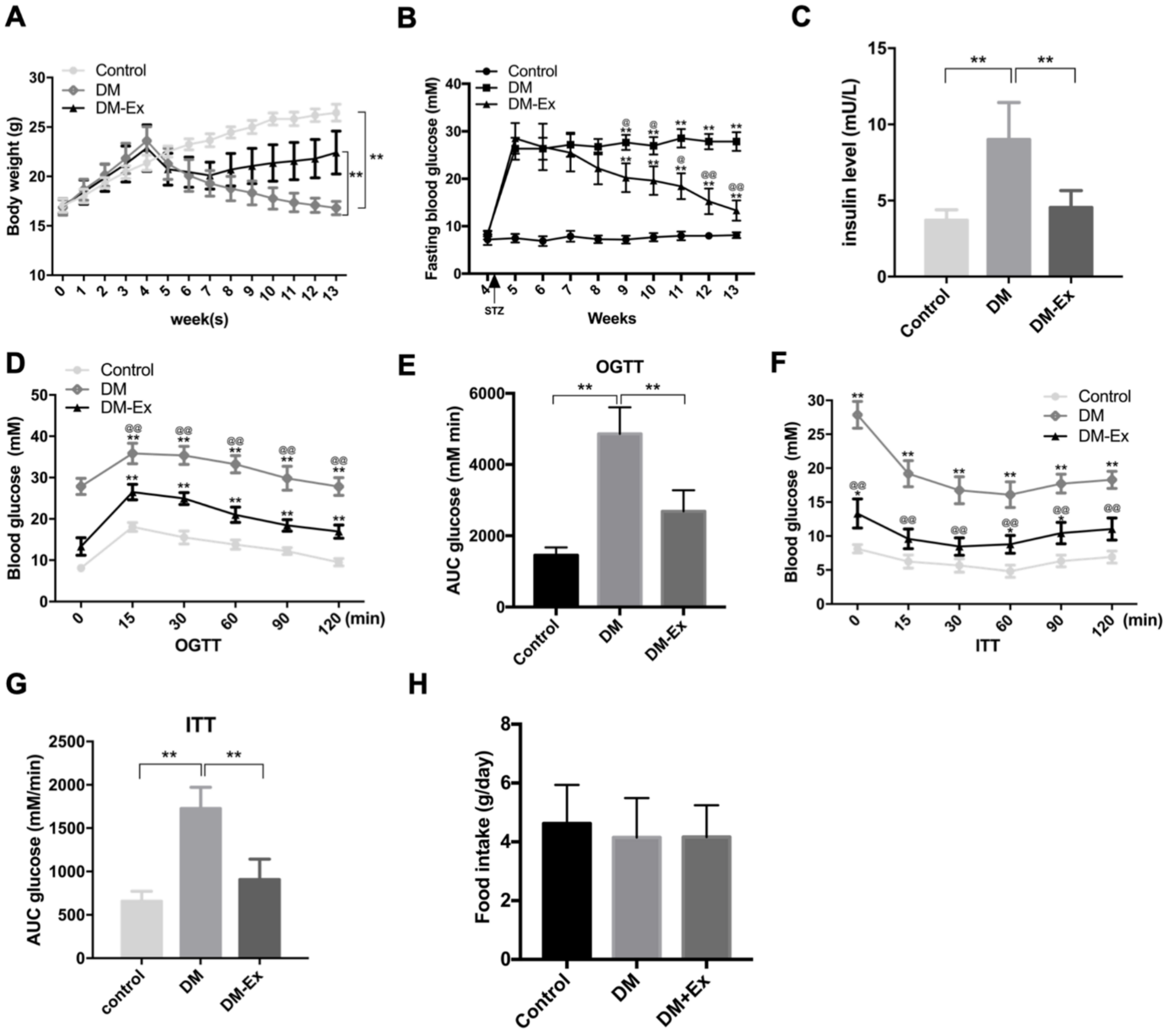

Figure S1. Indicator detection after establishment of T2DM model. (**A**) Body weight of mice in each group. (**B**) Fasting blood glucose in each group. (**C**) Insulin levels in mice of each group. (**D**) Glucose tolerance and (**E**) the area under glucose tolerance curve in each group. (**F**) Insulin tolerance and (**G**) the area under insulin tolerance curve in different groups. (**H**) Food intake in control mice and diabetic mice treated with or without exercise. * indicated the versus between control and DM. @ indicated the versus between DM+Ex and DM. * and @, $p < 0.05$. ** and @@, $p < 0.01$.

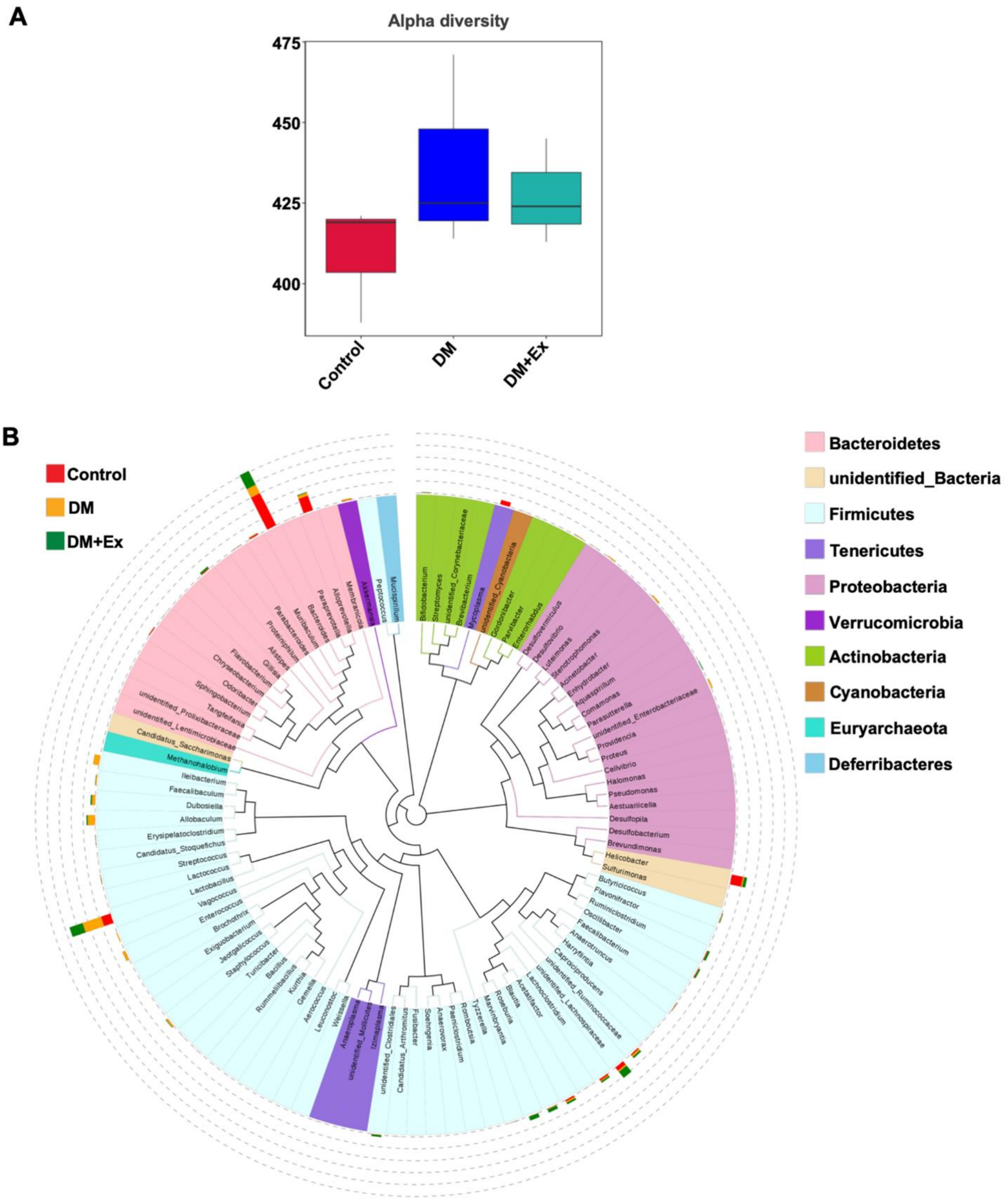

Figure S2. Analysis of intestinal flora sequencing results in each group. (**A**) Alpha diversity analysis. (**B**) Analysis of the genus-level species evolutionary tree results.

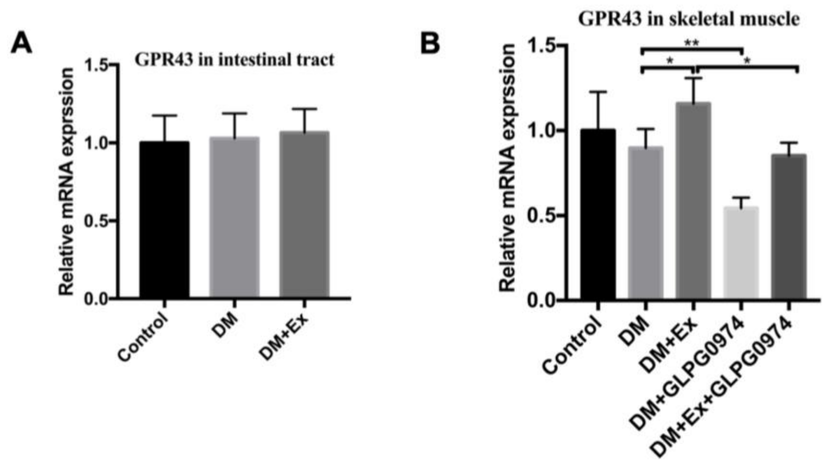

Figure S3. GPR43 expression in intestinal tract and skeletal muscle. (**A**) Detection of GPR43 expression in intestinal tract by RT-PCR. (**B**) Detection of GPR43 expression in skeletal muscle by ELISA. * $p < 0.05$. ** $p < 0.01$.

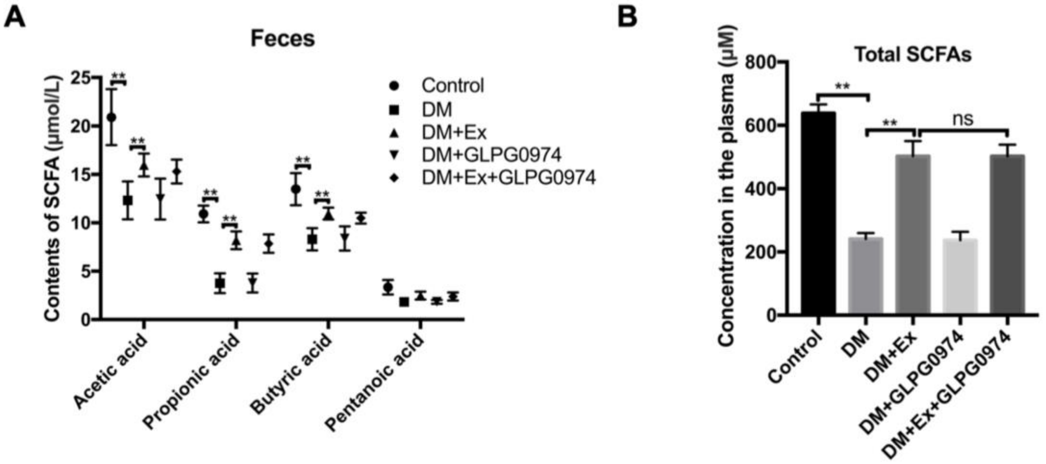

Figure S4. The addition of inhibitor GLPG0974 is independent of SCFA content in stool and plasma. (**A**) Detection of SCFAs content in stool of each group by GC. (**B**) Detection of total SCFAs concentration in plasma of mice by ELISA. * $p < 0.05$. ** $p < 0.01$.

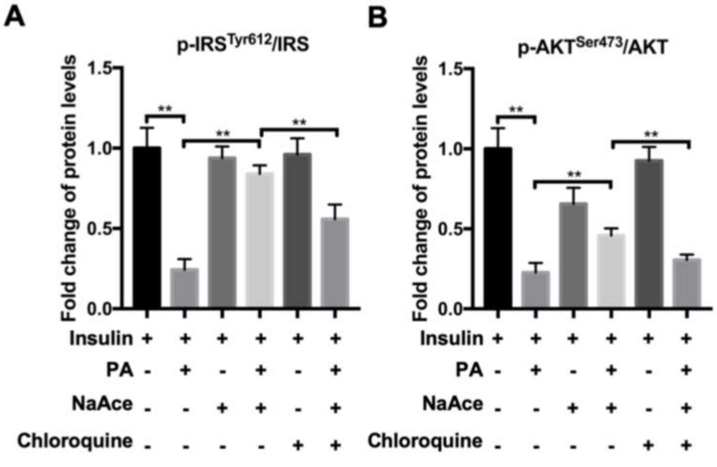

Figure S5. Quantitative analysis of protein levels in the Figure 5C. Semi-quantitative statistics of p-IRS^{Tyr612} (**A**) and p-AKT^{Ser473} (**B**) normalized to total IRS and AKT, respectively. ** $p < 0.01$.
